# Drug-induced burning mouth syndrome: a new clinico-pathological entity?

**DOI:** 10.1007/s10194-012-0486-x

**Published:** 2012-09-30

**Authors:** Giulio Fortuna, Annamaria Pollio

**Affiliations:** Oral Medicine Unit, Department of Odontostomatological and Maxillofacial Sciences, Federico II University of Naples, Via Pansini 5, 80131 Naples, Italy

Sir,

We read with great interest the letter by Coon et al. [1], recently published in this journal and we would like to focus our attention on the following concerns regarding this report:

1. Burning mouth syndrome is a chronic, idiopathic oral mucosal pain/discomfort, in which no clinical lesions or systemic diseases or other causes, especially drugs are identified [2, 3]. Some authors have used the term “secondary BMS” to indicate a form of oral complaint caused by local/systemic pathological conditions (including drugs), but even this term seems to be inappropriate, as the term “BMS” automatically excludes any known cause of any type of oropharyngeal complaint. Clinically, it is absolutely mandatory to differentiate between any oral burning, which can have many different causes (including drugs) and BMS, which describes a separate idiopathic clinico-pathological entity. Therefore, if this patient’s oral burning had been caused by carbidopa/levodopa, it would have been more appropriate to make a diagnosis of “carbidopa/levodopa-induced oral burning” and not “BMS or secondary BMS”.
2. If authors have suspected an adverse reaction to a specific drug, a reader might wonder why no one of the adverse drug reaction (ADR) algorithms [4, 5] has been used and no specific guideline [6] has been followed to conclusively prove a causative-relationship between oral burning sensation and carbidopa/levodopa.
3. In addition, one of these ADR algorithms would have helped clinicians in better differentiating between an oral burning induced by carbidopa/levodopa and an oral burning simply as a manifestation of Parkinson’s disease (PD). Indeed, considering the high association between PD and oral burning [7], PD might have caused this oral discomfort on its own.
4. Last, but not less important, it is necessary to distinguish an oral burning sensation as a clinical manifestation of an anxiety/depression trait or PD, considering either the association between PD and depressive symptoms (45 %) [8] and anxiety (50 %) [9] or between anxiety, depression and idiopathic BMS [10].

*Rebus sic stantibus*, it seems hard to establish where does this oral burning really come from (carbidopa/levodopa, PD, anxiety/depression, or a combination of these variables?) This case highlights, for all health care professionals, the need of a more rigorous diagnostic protocol before drawing any conclusion on a possible etiology of any oropharyngeal complaint.
